# Hemodynamic analysis of sequential graft from right coronary system to left coronary system

**DOI:** 10.1186/s12938-016-0259-x

**Published:** 2016-12-28

**Authors:** Wenxin Wang, Boyan Mao, Haoran Wang, Xueying Geng, Xi Zhao, Huixia Zhang, Jinsheng Xie, Zhou Zhao, Bo Lian, Youjun Liu

**Affiliations:** 1 College of Life Science and Bio-engineering, Beijing University of Technology, No.100 Pingleyuan, Chaoyang District, 100124 Beijing, China; 2 Beijing Anzhen Hospital Affiliated to Capital Medical University, Beijing, China; 3 Peking University People’s Hospital, Beijing, China

**Keywords:** Hemodynamics, Multi-branch, Sequential graft, Computational fluid dynamic (CFD)

## Abstract

**Background:**

Sequential and single grafting are two surgical procedures of coronary artery bypass grafting. However, it remains unclear if the sequential graft can be used between the right and left coronary artery system. The purpose of this paper is to clarify the possibility of right coronary artery system anastomosis to left coronary system.

**Methods:**

A patient-specific 3D model was first reconstructed based on coronary computed tomography angiography (CCTA) images. Two different grafts, the normal multi-graft (Model 1) and the novel multi-graft (Model 2), were then implemented on this patient-specific model using virtual surgery techniques. In Model 1, the single graft was anastomosed to right coronary artery (RCA) and the sequential graft was adopted to anastomose left anterior descending (LAD) and left circumflex artery (LCX). While in Model 2, the single graft was anastomosed to LAD and the sequential graft was adopted to anastomose RCA and LCX. A zero-dimensional/three-dimensional (0D/3D) coupling method was used to realize the multi-scale simulation of both the pre-operative and two post-operative models.

**Results:**

Flow rates in the coronary artery and grafts were obtained. The hemodynamic parameters were also showed, including wall shear stress (WSS) and oscillatory shear index (OSI). The area of low WSS and OSI in Model 1 was much less than that in Model 2.

**Conclusions:**

Model 1 shows optimistic hemodynamic modifications which may enhance the long-term patency of grafts. The anterior segments of sequential graft have better long-term patency than the posterior segments. With rational spatial position of the heart vessels, the last anastomosis of sequential graft should be connected to the main branch.

## Background

Veins from a certain part of the patient’s body are grafted to the coronary arteries in order to bypass atherosclerotic narrowing and enhance the blood supply to the coronary circulation supplying the myocardium [1]. Approximately 15–30% of saphenous vein graft (SVG) occluded within the first year of surgery, with the rate increasing to over 50% after 10 years [2]. Even if SVG failure still remain a significant clinical and economic burden, the majority of CABG options continue to use SVG [3]. Therefore, SVG was used in both models of this study. Some studies have shown that the long term patency of the sequential graft is obviously better than that of the single graft and the Y-type graft [4, 5]. Therefore, there were two models established in this study, adopting both the sequential graft and the single graft in each model, instead of using single grafts only.

There are three main coronary artery systems, which are RCA, LAD and LCX. The sequential graft is used alone among the different systems. Commonly, 50% of the blood supply to left ventricle (LV) is from LAD. In order to ensure the LV flow rate, the branches of LAD are usually used as the first anastomosis in clinical application. So did this research. But there are some doctors and researchers have come up with a question whether the sequential graft can be adopted between RCA and LAD, or between RCA and LCX. So in this study, two surgical options with both sequential graft and single graft, Model 1 and Model 2, were applied on a patient-specific model with multi-branch lesions. In Model 1, the anastomotic method was abbreviate as aorta (AOA)–LAD–LCX and AOA–RCA. In Model 2, the anastomotic method was abbreviate as AOA–RCA–LCX and AOA–LAD.

The 0D/3D coupling method was used in this numerical simulation to perform a numerical simulation by coupling the lumped parameter model (LPM; 0D sub-model) and 3D vascular sub-models. This method has been used in some new hemodynamic researches and proven useful in studying blood flow in the cardiovascular system [6–8].

The adaptive response to hemodynamic factors, i.e., WSS, may lead to SVG failure [9–11]. While, some studies show that hemodynamic factors are sensitive to the vascular geometry, small changes in the geometry may lead to severe changes on the distribution of hemodynamic factors [12, 13].

This work studies the hemodynamics of right coronary artery system anastomosis to left coronary system. In order to evaluate the novel multi-graft design (AOA–RCA–LCX and AOA–LAD), a computational fluid dynamics method was used to compare the novel grafts with the normal multi-graft (AOA–LAD–LCX and AOA–RCA) in terms of hemodynamic performance.

## Methods

### 3D reconstruction of patient anatomy and computational models

**Fig. 1:**
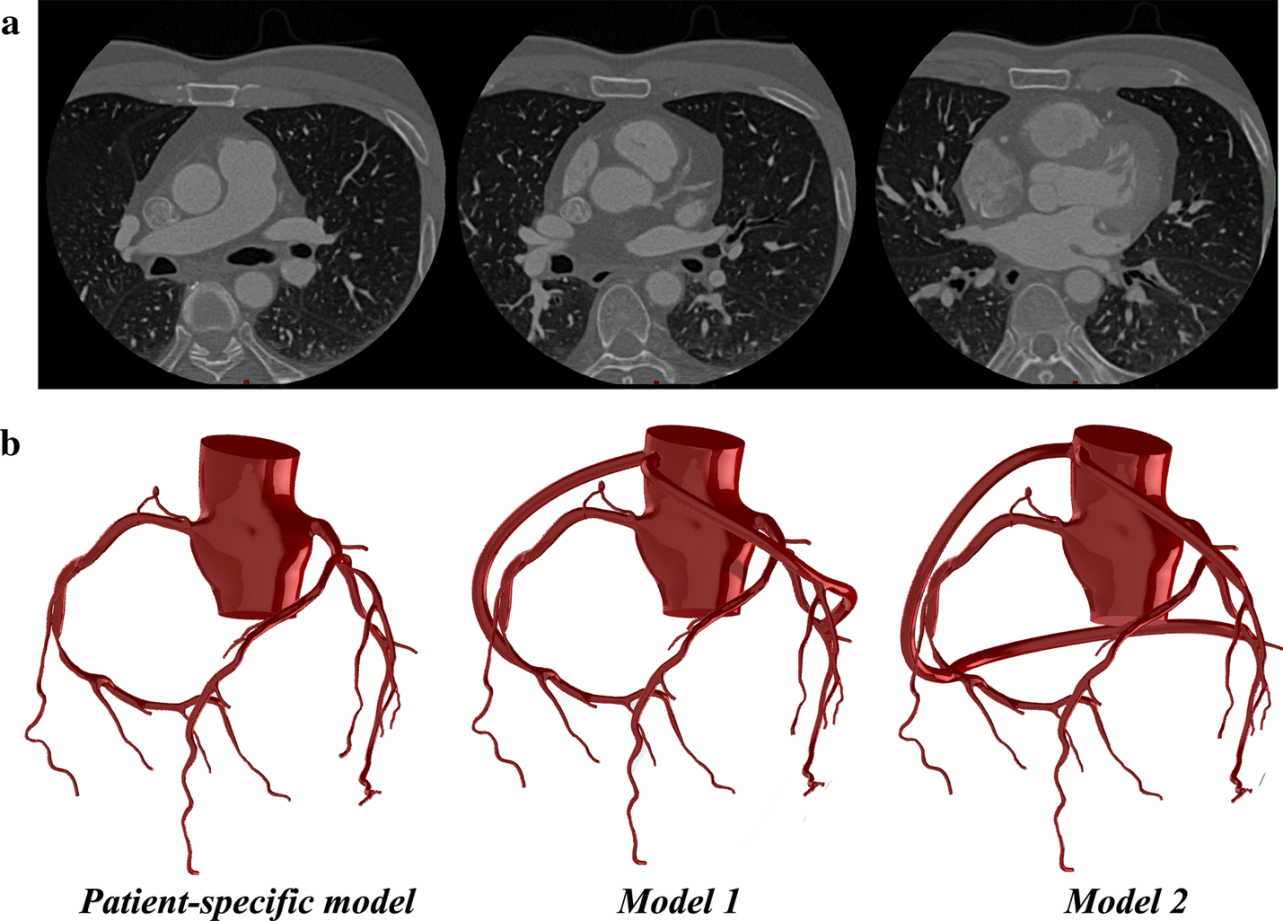
**a** CTTA images; **b** patient-specific model and two 3D models with different surgical options

In this study, a group of patient-specific anatomy data was acquired from a male patient with CAD obtained by Beijing Anzhen Hospital in China. Informed consent was obtained. The patient’s personal information was anonymized and de-identified prior to analysis. The cardiac output was 5.80 L/min, the systolic aortic pressure was 122.00 mmHg, and the diastolic aortic pressure was 84.00 mmHg. Two hundreds and seventy-one slices with 512 × 512 pixels are used for 3D reconstruction. The distance between each adjacent slice is 1 mm. Three slices of the patient’s CT images are shown in Fig. 1a. And the patient-specific 3D geometry of aorta and coronary artery tree was reconstructed as shown in Fig. 1b. Based on the method of virtual surgery, two postoperative 3D models were built by applying different sequential graft and single graft on this patient-specific model. In Model 1, the sequential grafting was from the LAD to the LCX, while the single grafting was anastomosis to the RCA. In Model 2, the sequential grafting was from the RCA to the LCX, while the single grafting was anastomosis to the LAD. The patient-specific model and two 3D models with different surgical options were shown in Fig. 1b.

The 3D sub-models were meshed to generate the computational models. Hexahedral mesh was generated mainly by using the size-control method with the assistance of the commercial software ANSYS-CFX (ANSYS™). The mesh was refined in the areas of interest to precise the simulation results as the resolution was improved. The number of nodes and elements in the two sequential models were within the same magnitude and all more than the number of computational requirements. A steady state grid sensitivity analysis was carried out to make sure that the number of the nodes and elements were large enough to ensure the stability and reliability of simulation results [14]. Solutions were obtained using a 1.97E6 element (1.52E6 node) finite element mesh in Model 1, while solutions were obtained using a 2.18E6 element (1.68E6 node) finite element mesh in Model 2.

In the 3D simulation the assumption of rigid wall was applied. The blood flow was treated as the incompressible viscous Newtonian fluid. The density of the blood flow was assumed to be 1.05 E3 kg/m^3^, and the dynamic viscosity was assumed to be 3.50 E-3 Pa· s.

### The 0D/3D coupled model

**Fig. 2:**
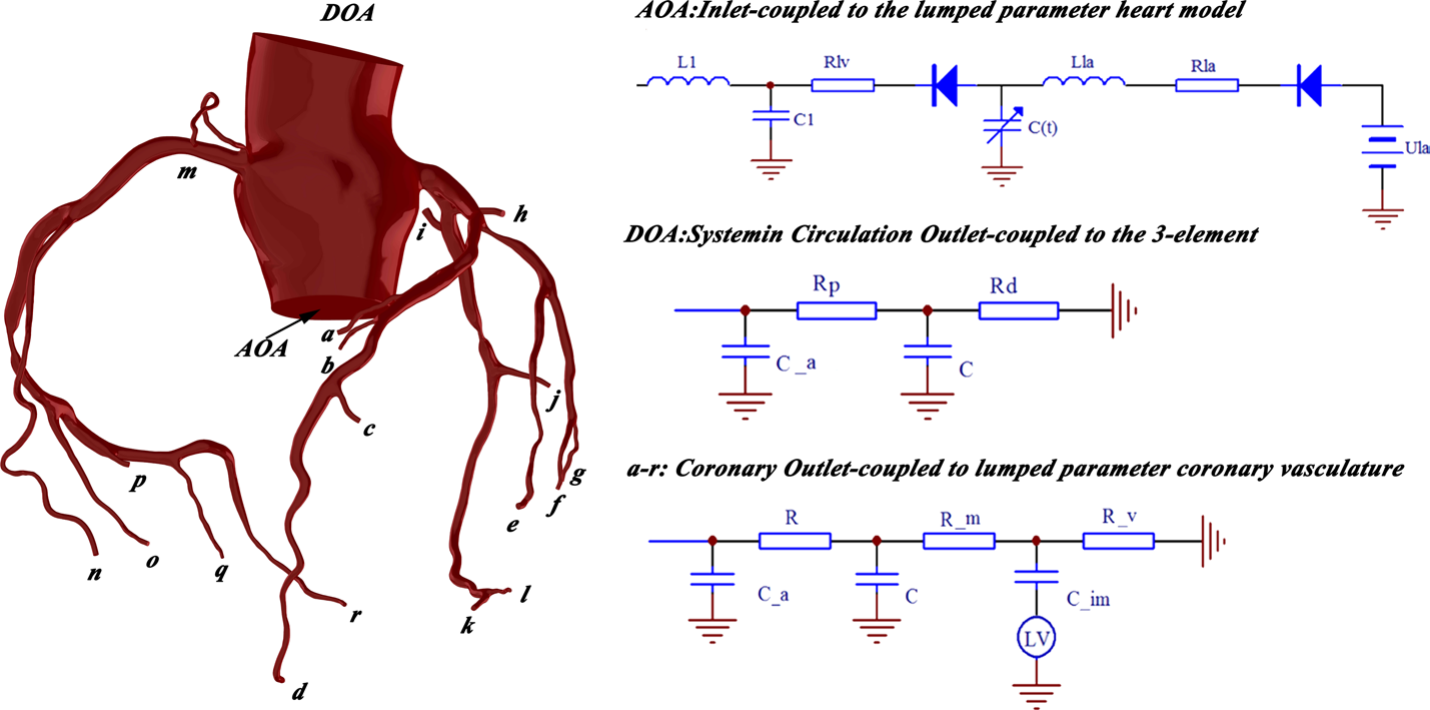
The 0D/3D coupled model

The LPM used in this study was referenced from Charles A. Taylor’s previous study [8, 14]. On the basis of the 3D vascular models and the patient’s cardiovascular system LPM, the 0D/3D coupled sequential coronary models were constructed as shown in Fig. 2. Since the patient’s peripheral vascular structure did not change, the two models shared the same 0D network. The 0D part of the coupled model (LPM) used in this study was referenced from our previous study [15]. In each block, the resistance (R) was used to simulate the flow resistance, the capacitance (C) was used to simulate the compliance of the vessel, and the inductance (L) was used to simulate the inertia of the blood flow.

In the compartment of the ventricle, the function of pressure–volume relationship was applied to demonstrate the cardiac cycle of the left and right ventricle.

1 $$E(t) = \frac{P(t)}{{V(t) - V_{0} }}$$

where *E*(*t*) is the time-varying elastance (mmHg/mL). *V*(*t*) and *P*(*t*) are the ventricle volume (mL) and pressure (mmHg) respectively. *V*_0_ is the reference volume (mL). Mathematically, the function was used as the approximation.

2 $$E(t) = (E_{\hbox{max} } - E_{\hbox{min} } ) \cdot E_{n} (t_{n} ) + E_{\hbox{min} }$$

where *E*_*n*_(*t*_*n*_) is the normalized time-varying elastance.

3 $$E_{n} (t_{n} ) = 1.55\left[ {\frac{{\left( {\frac{{t_{n} }}{0.7}} \right)^{1.9} }}{{1 + \left( {\frac{{t_{n} }}{0.7}}\right)^{1.9} }}} \right]\left[ {\frac{1}{{1 + \left( {\frac{{t_{n} }}{1.17}}\right)^{21.9} }}} \right]$$

*tc* is the cardiac cycle interval (s), $t_{n} = \frac{t}{{T_{\hbox{max} } }}$, *T*_max_ = 0.2 + 0.15 *tc*. In this paper, we set *E*_max_ = 2.0, *E*_min_ = 0.002458 and *tc* = 0.8 *s*.

The value of the parameters in the 0D network of coupled models were based on data from research into the modeling of coronary arteries [14]. The genetic algorithm was used to tune the parameters of the LPM model. In this way, the systolic pressure, the diastolic pressure and the cardiac output was matched the patient’s data. The list of model predictions and the clinical data are shown in Table 1. The tolerance error of data fitting was set to 1.72%.

**Table 1:** The list of model predictions and clinical data

	Clinical data	Model predictions
Systolic pressure (mmHg)	84.00	82.77
Diastolic pressure (mmHg)	122.00	119.92
Cardiac output (L/min)	5.80	5.90

## Results

### The aortic flow rate and pressure

**Fig. 3:**
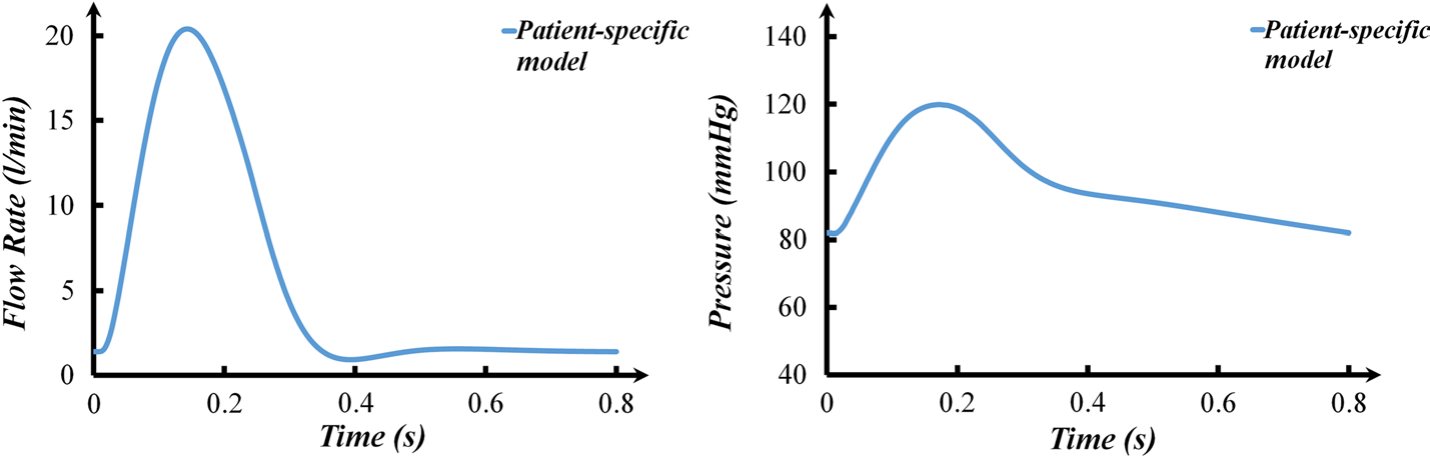
The aortic flow rate and pressure of the patient-specific model

The aortic flow rate and pressure of the patient-specific model were calculated as shown in Fig. 3. The cardiac output was 5.90 L/min, the systolic aortic pressure was 119.92 mmHg, and the diastolic aortic pressure was 82.77 mmHg. The difference between the numerical simulation results and clinical data was less than 2%.

### The coronary artery flow rate

**Fig. 4:**
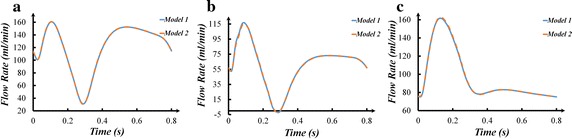
The flow rate: **a** LAD flow rate; **b** LCX flow rate; **c** RCA flow rate

**Fig. 5:**
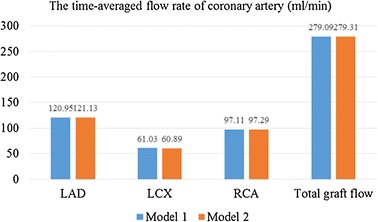
The time-averaged flow rate of coronary artery (mL/min)

The outlet flow rate of the LAD branches (a–g branches) was summed as the LAD flow rate. The outlet flow rate of the LCX branches (h–l branches) was summed as the LCX flow rate. The outlet flow rate of the RCA branches (m–r branches) was summed as the RCA flow rate. The coronary flow rate was illustrated in Fig. 4. It could be found that there were no significant difference in the LAD, LCX and RCA flow rate of two models. In addition, the time-averaged flow rate of coronary artery was calculated in Fig. 5.

### The graft flow

**Fig. 6:**
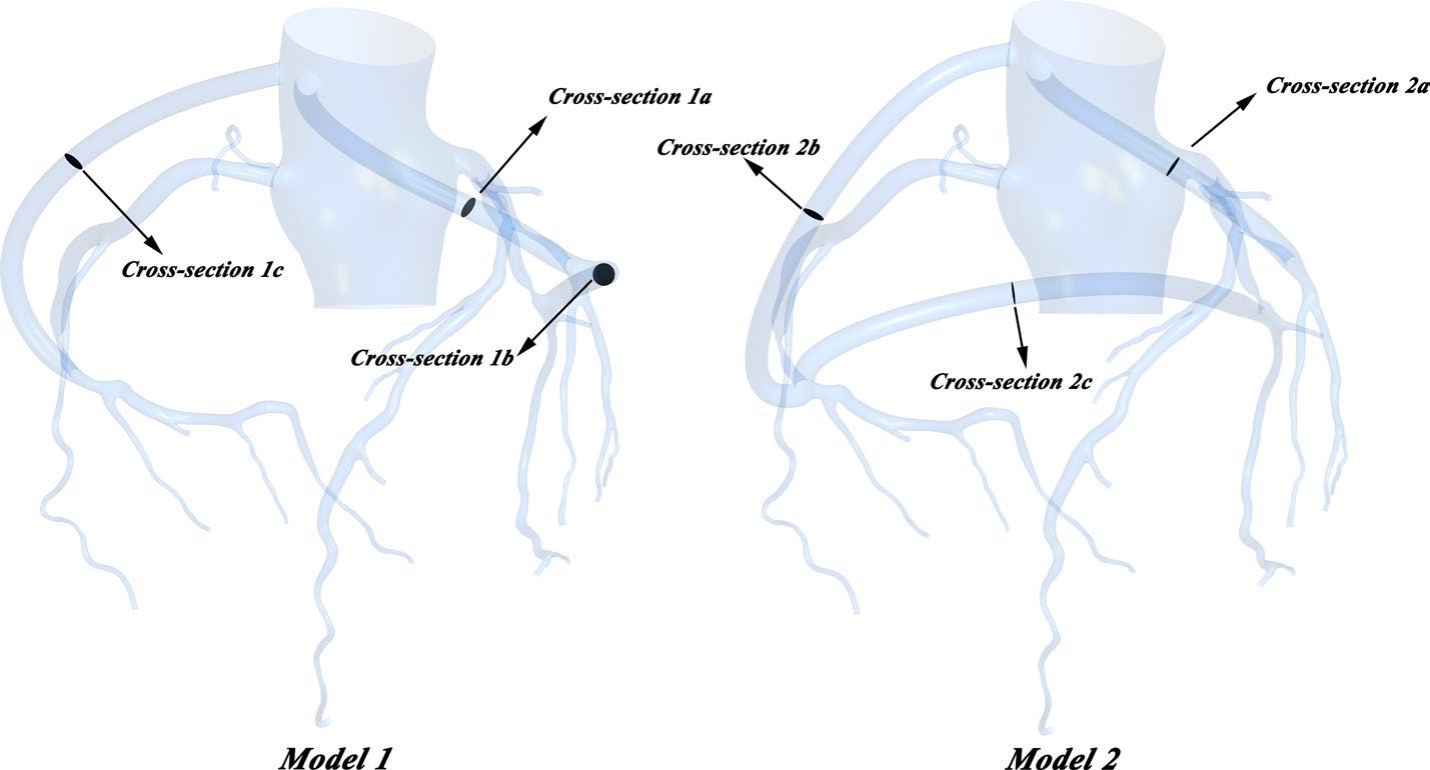
The six cross-sections used to calculate the graft flow rate

**Fig. 7:**
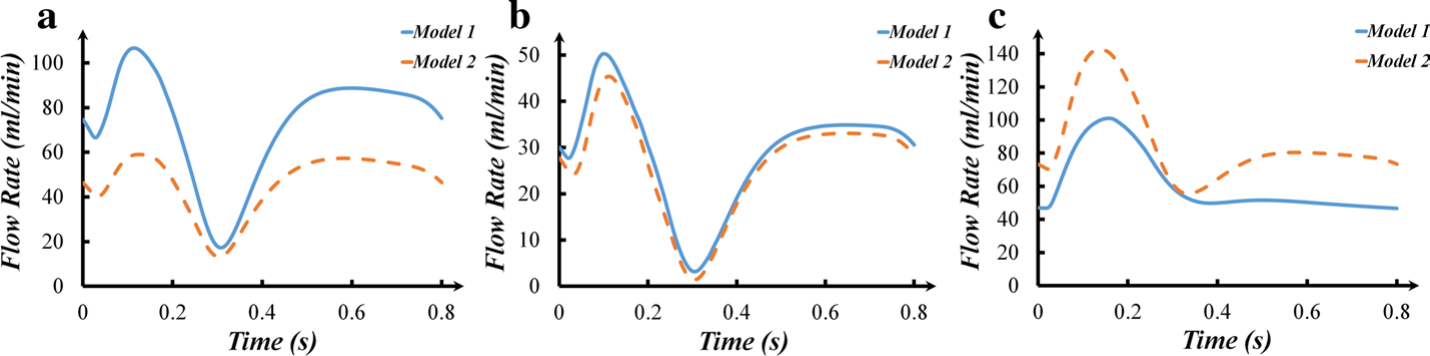
The flow rate: **a** graft to LAD; **b** graft to LCX; **c** graft to RCA

The graft flow was frequently used to evaluate the short-term outcomes of CABG [8]. In other words, it is the key indicator used by surgeons to assess the effectiveness of a graft. Six cross-sections on the grafts in the two models were picked out, as shown in Fig. 6. The flow rate through cross-section 1a and cross-section 2a was treated as the graft flow rate of LAD. The flow rate through cross-section 1b and cross-section 2c was treated as the graft flow rate of LCX. The flow rate through cross-section 1c and cross-section 2b was treated as the graft flow rate of RCA. The flow rate through each cross-section is depicted in Fig. 7.

**Fig. 8:**
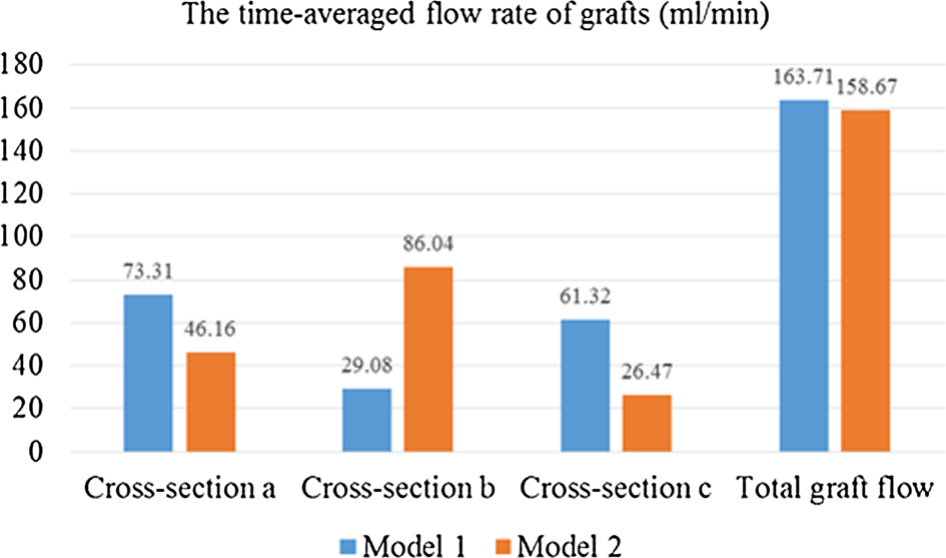
The area-average WSS of the grafts

Furthermore, the time-averaged flow rate of sequential graft and single graft were calculated in Fig. 8. In two models, the graft flow rate to LAD (cross-section 1a and 2a) was 73.31 and 46.16 mL/min respectively. While the graft flow rate to LCX (cross-section 1b, 2c) was 29.08 and 26.47 mL/min respectively. The graft flow rate to RCA (cross-section 1c, 2b) was 61.32 and 86.05 mL/min respectively.

### The wall shear stress

**Fig. 9:**
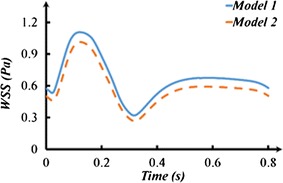
The WSS contour at the extreme value time

**Fig. 10:**
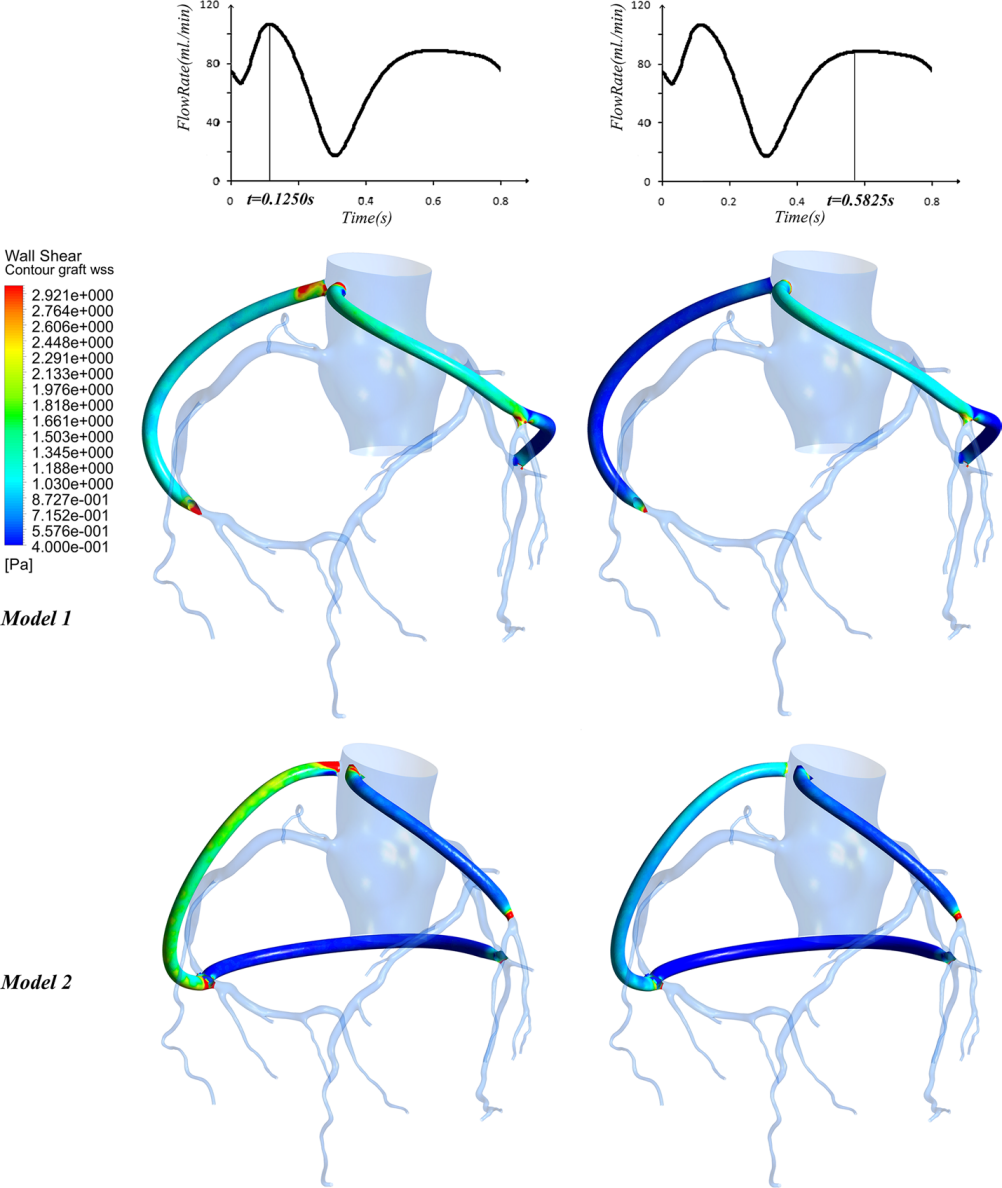
The time-averaged graft flow rate (mL/min) through every cross-section

Wall shear stress (WSS) of the grafts in two models are plotted. The waveforms of area-averaged WSS on the grafts are illustrated in Fig. 9. The time-averaged values of theses waveforms was calculated according to the expression in Eq. (4). The TAWSS for Model 1 and Model 2 are 0.67 and 0.59 respectively. Moreover, when flow rate of the grafts got the maximum (t = 0.13 and 0.58 s), the WSS distributions are shown in Fig. 10. Furthermore, the area and percentage of low WSS (≤0.4 Pa) in two models have been calculated and listed in Table 2. In Model 1, the area of low WSS was 68.37 mm^2^ (t = 0.13 s) and 433.35 mm^2^ (t = 0.58 s). While in Model 2, the area of low WSS was 86.30 mm^2^ (t = 0.13 s) and 1250.04 mm^2^ (t = 0.58 s). It could be found that the low WSS area of grafting in Model 1 was obviously smaller than that in Model 2 at 0.58 s.

**Table 2:** The area and percentage of low WSS on the graft

Model (mm^2^)	Area (mm^2^)		Percentage (%)	
	t = 0.13	t = 0.58	t = 0.13	t = 0.58
Model 1 (2647.36)	68.37	433.35	2.58%	16.37%
Model 2 (3424.10)	86.30	1250.04	2.52%	36.51%

4 $${\text{TAWSS}} = \frac{1}{T}\mathop{\int }\nolimits_{0}^{\text{T}} \left| {\overrightarrow {{{{\tau }}_{{{\omega }}} }} } \right|{\text{dt}}$$

### The oscillatory shear index

**Fig. 11:**
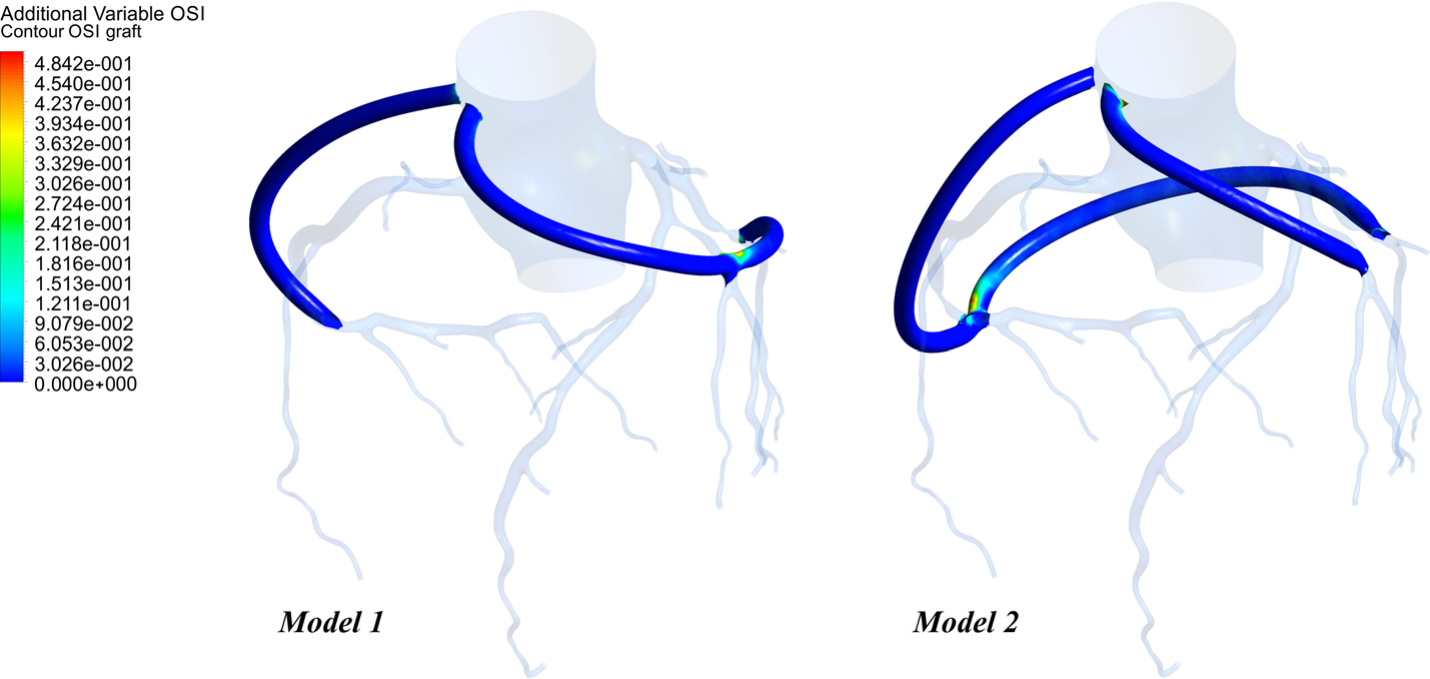
The OSI contour on the grafts

**Fig. 12:**
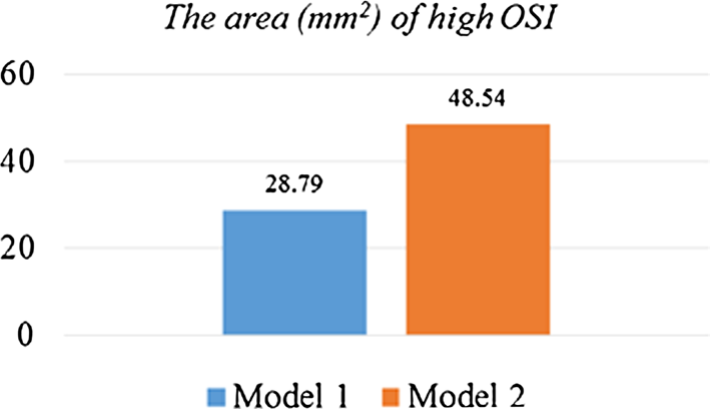
The area of high OSI

Oscillatory shear index (OSI) is a measure of the temporal and spatial variations of local WSS. The OSI was calculated according to the expression in Eq. (5). The values of OSI range from 0 in undisturbed flow with unidirectional shear stress vectors to 0.5 in disturbed flow with oscillatory shear stress vectors. The OSI contour on the grafts of two models were illustrated in Fig. 11. It could be found that the high OSI values only appeared at a few localized anastomosis regions in two models. The area of high OSI (≥0.15) on the grafts was listed in Fig. 12. Moreover, the area-average OSI of the grafts in two models are calculated. It can confirm that the OSI in Model 1 is higher than that in Model 2.

5 $${\text{OSI}} = 0.5 \times \left( {1 - \frac{{|\mathop {\int }\nolimits_{0}^{\text{T}} {{\tau }}_{{{\omega }}} {\text{dt}}|}}{{\mathop {\int }\nolimits_{0}^{\text{T}} \left| {{{\tau }}_{{{\omega }}} } \right|{\text{dt}}}}} \right)$$

## Discussion

### Clinical significance

#### Short-term outcomes

Flow rate of grafting was frequently used to evaluate the short-term outcomes of CABG. From the results, it could be found that there was no significant difference in flow rate of coronary artery between two models. This phenomenon meant that the sequential graft and the single graft of two models did not change the flow distribution to every coronary artery branches. The flow rate distribution of every branch was mainly controlled by the outlet pressure and the resistance. In terms of the blood supply mechanism, the sequential graft was feasible from RCA branch to LCX branch.

#### Long-term patency

The different multi-grafts options would affect the long-term patency of graft. Some researches declared that low WSS might decrease the long-term patency of the graft [16, 17] or lead to SVG failure [10, 11]. In Model 1, the area of low WSS was 433.35 mm^2^ (t = 0.58 s), while in Model 2, the area of low WSS was 1250.04 mm^2^. The low WSS area of grafting in Model 1 was obviously smaller than that in Model 2 at 0.5280 s. It could be found that the low WSS region mainly appeared on the sequential graft to the LCX.

If we assumed that the diameter of sequential grafts was constant, the WSS was proportional to the velocity of flow. So we hold that (i) when the flow rate of cross-section 1b and 2c was the lowest in two models, the WSS of graft to LCX was smallest; (ii) the anterior segments of sequential graft could have better long-term patency than the posterior segments.

Some study indicated that lower OSI would decrease the opportunity of vascular intimal hyperplasia [18, 19]. High OSI values were considered to be the crucial factors that led to the functional incapacitation of intimal cells and the change of cell structure. It could be found that high OSI area existed in both grafts, especially at the middle anastomosis as shown in Fig. 11. The area of high OSI (≥0.15) on the grafts was calculated. In Model 1, the area of high OSI was 28.79 mm^2^, while the area of high OSI was 48.54 mm^2^ in Model 2. Moreover, the area-average OSI of the grafts in Model 1 and 2 was 7.99 E–3 and 1.18 E–2 respectively. Therefore, the long-term patency of Model 1 was better than that of Model 2.

#### The universality of conclusions

This paper, based a patient-specific model, is to clarify the possibility of right coronary artery system anastomosis to left coronary system. But it can’t prove that the conclusions have universality to other patients.

### Future work

Fractional flow reserve (FFR) is an important index of coronary stenosis, isolates a specific period in diastole, called the wave-free period. For one patient, the value of FFR decrease with the Stenosis degree. But different patient may have different critical stenosis degree result in myocardial ischemia and low FFR. Therefore, the relationship among stenosis degree, FFR and sequential graft from left coronary system to right coronary system is also an interesting research content which may be studied in the future.

### Limitation

Some limitations existed in this study. First, the rigid wall hypothesis was applied in the 3D simulation. The fluid–structure interaction was not used because it would cost much time in the 3D calculation. Second, there were only sequential graft and single graft considered in two models, since majority studies proposed that sequential graft was better than the other type graft. The results and conclusions might be different for other surgical procedures or stenosis.

## Conclusion

It is feasible that right coronary artery system anastomosis to left coronary system. Two post-operative models with different multi-grafts options were built to discuss the short-term outcomes and long-term patency by investigating the hemodynamic effects based on the 0D/3D coupling simulation. There were no significant differences in the short-term outcomes on two models which met the demand of patient flow supply. But the surgical method of Model 2 had more areas of low WSS and high OSI. Therefore, Model 2 had better long-term patency than Model 1.

We come to the conclusion that (i) a branch of LAD also can be the last anastomosis in sequential graft; (ii) when the spatial position of the heart vessels is permitted, the last anastomosis of the sequential graft should reach to the main branch with large flow demand.

## Abbreviations

CHD: coronary heart disease
CCTA: coronary computed tomography angiography
RCA: right coronary artery
LAD: left anterior descending
LCX: left circumflex artery
0D/3D: zero-dimensional/three-dimensional
WSS: wall shear stress
OSI: oscillatory shear index
CFD: computational fluid dynamic
CABG: coronary artery bypass grafting
SVG: saphenous vein graft
LV: left ventricle
LPM: lumped parameter model

## References

[1] Bravata DM, Gienger AL, McDonald KM, Sundaram V, Perez MV, Varghese R (2007). Systematic review: the comparative effectiveness of percutaneous coronary interventions and coronary artery bypass graft surgery. Ann Intern Med.

[2] Mehta D, Izzat MB, Bryan AJ, Angelini GD (1997). Towards the prevention of vein graft failure. Int J Cardiol.

[3] Bryan AJ, Angelini GD (1994). The biology of saphenous vein graft occlusion: etiology and strategies for prevention. Curr Opin Cardiol.

[4] Hajati O, Zarrabi K, Karimi R, Hajati A (2012). The biology of saphenous vein graft occlusion: etiology and strategies for prevention. IEEE Eng Med Bio.

[5] Zhao X, Liu YJ, Wang WX (2015). Hemodynamic based surgical decision on sequential graft and Y-type graft in coronary artery bypass grafting. Mol Cell Biomech..

[6] Lagana K, Balossino R, Migliavacca F, Pennati G, Bove EL, de Leval MR (2005). Multiscale modeling of the cardiovascular system: application to the study of pulmonary and coronary perfusions in the univentricular circulation. J Biomech.

[7] Hsia TY, Migliavacca F, Pennati G, Balossino R, Dubini G, de Leval MR, Bradley SM, Bove EL (2009). Management of a stenotic right ventricle-pulmonary artery shunt early after the Norwood procedure. Ann Thorac Surg.

[8] Taylor CA, Fonte TA, Min JK (2013). Computational fluid dynamics applied to cardiac computed tomography for noninvasive quantification of fractional flow reserve: scientific basis. J Am Coll Cardiol.

[9] Huo Y, Luo T, Guccione JM, Teague SD, Tan W, Navia JA (2013). Mild anastomotic stenosis in patient-specific CABG model may enhance graft patency: a new hypothesis. PLoS ONE.

[10] Leask RL, Butany J, Johnston KW, Ethier CR, Ojha M (2005). Human saphenous vein coronary artery bypass graft morphology, geometry and hemodynamics. Ann Biomed Eng.

[11] Butany JW, David TE, Ojha M (1998). Histological and morphometric analyses of early and late aortocoronary vein grafts and distal anastomoses. Can J Cardiol.

[12] Fenton KN, Siewers RD, Rebovich B, Pigula FA (2003). Interim mortality in infants with systemic-to-pulmonary artery shunts. Ann Thorac Surg.

[13] Pennati G, Fiore GB, Migliavacca F, Lagana K, Fumero R, Dubini G (2001). In vitro steady-flow analysis of systemic-to-pulmonary shunt haemodynamics. J Biomech.

[14] Kim HJ, Vignon-Clementel IE, Coogan JS, Figueroa CA, Jansen KE, Taylor CA (2010). Patient-specific modeling of blood flow and pressure in human coronary arteries. Ann Biomed Eng.

[15] Zhao X, Liu YJ, Li LL, Wang WX, Xie JS, Zhao Z (2016). Hemodynamics of the string phenomenon in the internal thoracic artery grafted to the left anterior descending artery with moderate stenosis. J Biomech.

[16] Corban MT, Piccinelli M, Timmins LH, Passerini T, Eshtehardi P, Nanjundappa RA (2012). Lower coronary wall shear stress is associated with endothelial dysfunction in patients with non-obstructive coronary artery disease. Circulation.

[17] Davies PF, Civelek M (2011). Endoplasmic reticulum stress, redox, and a proinflammatory environment in athero-susceptible endothelium in vivo at sites of complex hemodynamic shear stress. Antioxid Redox Sign..

[18] Weintraub WS, Jones EL, Morris DC, King SB, Guyton RA, Craver JM (1997). Outcome of reoperative coronary bypass surgery versus coronary angioplasty after previous bypass surgery. Circulation.

[19] Zhang C, Xie S, Li SY, Pu F, Deng XY, Fan YB (2012). Flow patterns and wall shear stress distribution in human internal carotid arteries: the geometric effect on the risk for stenoses. J Biomech.

